# Red seaweed supplementation suppresses methanogenesis in the rumen, revealing potentially advantageous traits among hydrogenotrophic bacteria

**DOI:** 10.1186/s40168-025-02251-2

**Published:** 2025-11-13

**Authors:** Pengfan Zhang, Breanna Roque, Pedro Romero, Nicole Shapiro, Emiley Eloe-Fadrosh, Ermias Kebreab, Spencer Diamond, Matthias Hess

**Affiliations:** 1 Innovative Genomics Institute, University of California, Berkeley, CA USA; 2 Department of Animal Science, University of California, Davis, CA USA; 3 FutureFeed, Townsville, QLD Australia; 4 DOE Joint Genome Institute, Berkeley, CA USA

**Keywords:** Enteric methane, Microbial ecology, Microbiome, Rumen

## Abstract

**Background:**

Macroalgae belonging to the genus *Asparagopsis* have shown to reduce methane (CH_4_) production during rumen fermentation, while increasing feed efficiency when added to the feed of cattle. However, little is known about how the rumen microbiome responds to *Asparagopsis* supplementation, and how changes in the microbiome may contribute to changes in rumen function and host phenotype. Here, we generated and analyzed metagenomic and metatranscriptomic data from the rumen microbiome from cows receiving (treatment) and not receiving (control) an *Asparagopsis armata* supplemented diet.

**Results:**

Using a combination of metatranscriptome and metagenome analysis, we found that reduction of CH_4_ emission from animals receiving *A*.* armata* was coupled to a significant reduction in the transcription of methanogenesis pathways. Additionally, a significant decrease in the transcription of genes for carbon catabolism and a reorganization of carbon catabolic gene expression occurred at the species level within the rumen microbiome of animals that received red seaweed with their diet. Increased H_2_ production, a consequence of methanogenesis suppression, was coupled to a significant increase in the transcription of hydrogenases that mediate hydrogenotrophic metabolism in the treatment group. Metatranscriptome analysis identified a single metagenome assembled genome (MAG) of a *Duodenibacillus* sp., a hitherto uncultured hydrogenotrophic bacterial species, as the dominant driver of this transcriptional change.

**Conclusions:**

Comparative genomic analysis between the *Duodenibacillus *sp. and other hydrogenotrophic rumen organisms revealed metabolic traits that may provide *Duodenibacillus* sp. with a competitive advantage in H_2_ scavenging. Our findings provide an initial understanding of how the rumen microbiome responds to a promising CH_4_ reducing feed additive and serve as a model for alternative stable rumen microbiome states that produce less methane and increase animal productivity. Ultimately, insights from the work presented here might enable the development of advanced microbiome-based strategies to reduce enteric methane production.

**Supplementary Information:**

The online version contains supplementary material available at 10.1186/s40168-025-02251-2.

## Background

Methane, a potent greenhouse gas, exhibits a global warming potential 28 times greater than that of carbon dioxide (CO_2_) over a 100-year period [1]. Ruminant livestock are a major source of methane (CH_4_), contributing nearly 30% of anthropogenic CH_4_ emissions both in the USA and worldwide [2]. As the demand for ruminant-sourced products continues to rise [3], more effective and sustainable approaches to reduce CH_4_ emissions from this sector are needed.

Enteric methane is a waste product of the anaerobic fermentation process of the animal feed, facilitated by a complex consortium of bacteria, fungi, and protozoa [4–8]. The rumen microbiome is crucial for the animal’s health and survival due to its key role in generating volatile fatty acids (VFAs), which supply up to 70% of the animal’s energy requirements [9]. Alongside VFAs, this fermentation process also yields substantial amounts of hydrogen (H_2_) and carbon dioxide (CO_2_) [6]. In the terminal stages of rumen fermentation, excess H_2_ is predominantly eliminated by archaeal methanogens, with a minor contribution from VFA-producing hydrogenotrophic nitrate- and sulfate-reducing bacteria [10]. Despite their relatively low abundance, rumen methanogens serve as the most important electron sink, propelling the fermentation process forward [8, 10]. Additionally, methanogens influence the overall pH of the rumen, which affects some of the major fibrolytic rumen bacteria [11]. Consequently, the hydrolysis of biomass impacted by methanogen activity, and a notable correlation exists between the abundance of methanogens and cellulolytic microorganisms [12]. Despite their importance as electron utilizers and their broader influences on the rumen microbiome, suppression of methanogens does not always negatively impact rumen functionality [13]. While the various contributions of methanogens could potentially be filled by other naturally occurring rumen microorganisms [14, 15], there is limited data on alternative rumen microbiome states where methanogens are inhibited. This represents a knowledge gap, as the inhibition of methanogenesis could free reducing equivalents for the production of VFAs [16–20], which in turn would be available for utilization by the host animal, thereby increasing the ruminant’s feed efficiency and productivity [16, 19].

Numerous strategies have been explored to curb CH_4_ emissions from ruminants, including the introduction of biological and synthetic feed additives [16, 19, 21–25], dietary adjustments [26–28], and selective breeding [29, 30]. Although selective breeding over several generations has shown to be effective, there is a limitation on the extent it can yield further improvements to curb climate change [31]. Feed additives and dietary modifications offer more immediate avenues for intervention, yet some of them adversely affect VFA production or overall animal productivity [32]. Members of the genus *Asparagopsis*, a red seaweed that produces and stores various halogenated CH_4_ analogues [32], have emerged as a promising biological solution for significantly reducing CH_4_ emissions from ruminants [19, 23, 32, 33]. Comprehensive reviews detailing the potential of seaweeds to reduce enteric methane, including some key considerations for their global application can be found in McGurrin et al. [34] and Vijn et al. [35].

Previous work has shown that *Asparagopsis *sp. can cut methane emissions by up to 99% during in vitro rumen fermentation without negatively impacting VFA profiles or feed digestibility [32, 36]. In vivo studies further validate these findings, showing CH_4_ reductions of up to 67% in dairy cows [19] and up to 98% in beef steers [37]. Notably, the methane-lowering effect of *Asparagopsis* persisted over a prolonged animal trial of 21 weeks and enhanced feed efficiency [38]. This suggests a potential metabolic shift within the rumen microbiome that allowed animals to generate energy precursors without the accompanying methane production.

It has been hypothesized that *Asparagopsis *sp. exert their anti-methanogenic properties likely through the activity of bromoform, or a bromoform derivative, and its capability to bind and inhibit coenzyme F430, an essential cofactor in methanogenesis [39, 40]. However, the potency of *Asparagopsis *sp. may stem from the synergistic action of multiple bioactive metabolites present in the algal biomass, targeting various reactions within the methanogenesis pathway, and therefore exceeding the methane-reducing effect of pure bromoform [41]. While *Asparagopsis *sp. has shown promise for reducing enteric methane, a detailed response of the rumen microbiome, such as microbial shifts, methanogenesis regulation, and transcriptional dynamics, to *Asparagopsis* supplementation remain poorly understood.

To address this knowledge gap, we performed genome-resolved metagenomic and metatranscriptomic analyses of the microbiome associated with rumen fluid from 8 animals at the end of an *Asparagopsis armata* feeding trial [19]. In this trial, a total of 12 animals received feed with and without *A*. *armata* over 14 days following a 3 × 3 Latin square experimental design with 4 animals per treatment group (*n* = 12 animals), similar to an earlier study that investigated the metagenomic response of low and high methane emitting sheep [42]. The experimental design for the animal trial and the time point for rumen fluid collection is illustrated in Fig. S1. The multi-omics data, generated and analyzed for the work presented here, revealed qualitative shifts in the rumen microbiome, notably a decrease in methanogenic archaea in animals that received *A*. *armata* supplemented feed. Furthermore, metatranscriptomic data indicated significant transcriptional reprogramming within the microbiomes of these animals, including (i) a widespread suppression of genes known to be involved in methanogenesis, (ii) an upregulation of hydrogenotrophic pathways, and (iii) a decrease in the expression of genes associated with complex carbohydrate degradation. Together, these data support the capability of *A*. *armata*, and most likely of *Asparagopsis *sp. in general, to suppress genes across at least two major methanogenesis pathways and provide insight into the host phenotype that has been described in detail previously [19]. Whereas findings from our work provide new insights into the mechanisms by which methanogenesis suppression may reorganize rumen microbiome composition and functions toward states that might be more beneficial for the environment and the productivity of the animal, additional work with more animals and over extended time will be needed before conclusions regarding the general response of the rumen microbiome response to *A*. *armata* across different ruminant species and feeding systems can be made. Nonetheless, an enhanced understanding of these functional shifts at the genome and gene level, as provided by the work presented here, will be essential to identify organisms and pathways that drive these functional shifts and to enable the development of next generation rumen modulation approaches.

## Results

### An integrated rumen microbiome database enhances species profiling

**Fig. 1:**
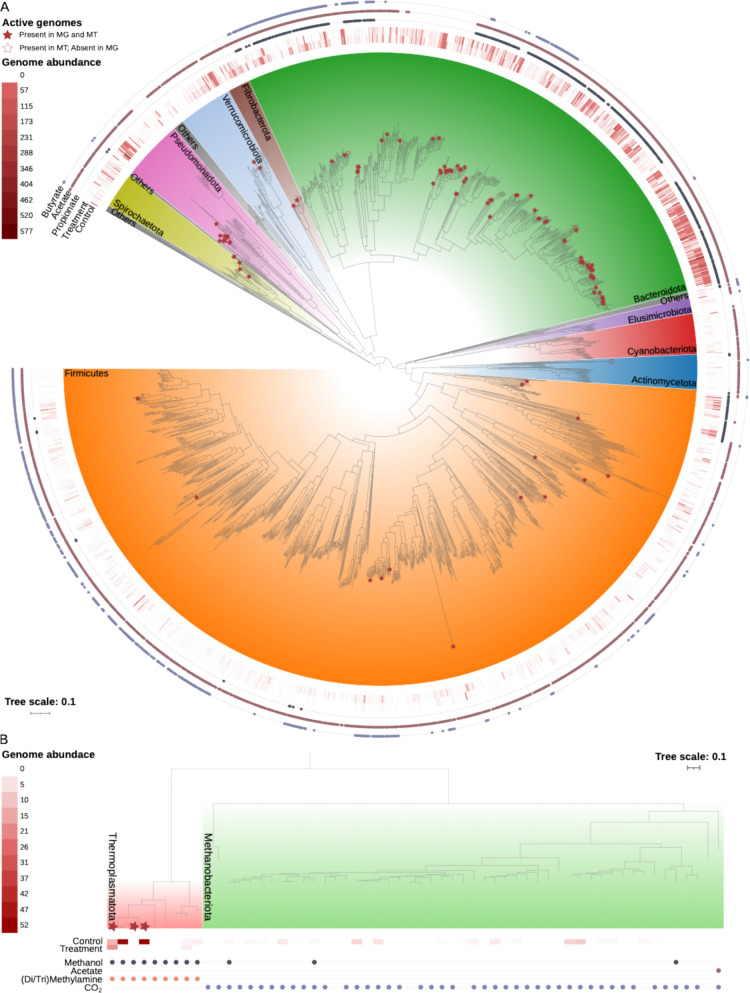
Phylogenetic tree of detected rumen bacteria and archaea. **A** Phylogenetic tree of 1949 bacterial RuMAGs detected either in metagenomic or metatranscriptomic samples of this study (*n* = 2013 detected in total). Tree was constructed using a concatenated set of bacterial-specific marker genes, and rooted at the midpoint. Some species detected in metagenomic or metatranscriptomic samples were omitted from the tree due to insufficient numbers of marker genes. Colored ranges over tree branches indicate phylum level taxonomy. Phyla that had fewer than 20 species were consolidated in “Others.” Solid stars indicate RuMAGs which were detected in metagenomic samples and were also considered transcriptionally active (*n* = 99 genomes; see “Methods” section). Non-solid stars indicate RuMAGs that were transcriptionally active, but were not detected in metagenome samples (*n* = 11 genomes; see “Methods” sction). The heatmap shows normalized RuMAG abundance within the metagenome samples as indicated in the inner two rings (for control and treatment samples). The outer three rings indicate the presence of VFA (e.g., propionate, acetate, and butyrate) biosynthetic pathways in genomes by the presence of colored circles. **B** Phylogenetic tree of all 58 archaeal RuMAGs from the methanogenic phyla *Thermoplasmatota* and *Methanobacteriota* in the rumen-specific database created for this study. Tree was constructed using a concatenated set of archaeal-specific marker genes and rooted at the midpoint. Solid stars indicate RuMAGs which were detected in metagenomic samples and were also considered transcriptionally active (*n* = 3 genomes; see “Methods” section). The heatmap shows normalized RuMAG abundance within the metagenomes as indicated in the first two lines (for control and treatment samples). Colored points below the heatmap indicate the presence in RuMAGs of methanogenic metabolic pathways that can utilize different starting substrates. For phylogenetic trees showing all RuMAGs in the rumen specific database created for this study see Fig. S2

Rumen fluid was collected from 8 lactating Holstein cows following 14 days of feed supplementation with (*n* = 4 animals) and without (*n* = 4 animals) *Asparagopsis armata* at an inclusion rate of 1% [19] (Fig. S1). Cows treated with *A*. *armata* exhibited a decrease in methane production of up to 60%, a 367% increase in hydrogen production, and an increase in feed efficiency of up to 74%. Detailed phenotypic responses of the animals to *A. armata* supplementation were measured and reported by Roque et al. [19]. For the reader’s convenience, these data are summarized and included in Table S1. To recover microbial genomes specifically found in the rumen of animals sampled during this study, we extracted total microbial DNA from the rumen of one cow from each treatment group and generated ~ 30 Gb of shotgun sequence data per sample for subsequent metagenomic analysis (Table S2). Assembly and binning of metagenomic reads from these samples resulted in the recovery of 72 draft genome bins at the species-level (ANI ≥ 95%) each with estimated completeness ≥ 60% and contamination ≤ 10%. We also generated a total of 400 Gbp (~ 50 Gb/sample) of metatranscriptome data from all 8 animals in the study (Table S2). To increase the probability that metagenomic and metatranscriptomic reads could be assigned to microbial genomes, we combined the 72 draft genomes recovered in this study with rumen associated isolate genomes and rumen metagenome-assembled genomes (RuMAGs) from three public datasets [43–45] to create a comprehensive cohort-specific rumen genome database. Initially, this integrated genome database contained 547 genomes from microbial isolates and 10,591 RuMAGs. After these genomes and RuMAGs were de-replicated at the species level (ANI ≥ 95%), our database contained genomes from 3119 bacterial and 61 archaeal species across 27 phyla, with 35 species exclusively recovered from this study (Fig. 1,Fig. S2, and Table S3). Hereafter, we refer to the 3180 species-level representative genomes that encompass RuMAGs and isolate assembled genomes as RuMAGs. Our RuMAG database yielded significantly higher mapping rates for metagenomic samples relative to any of the individual rumen-specific databases alone (Table S4). Furthermore, we compared the efficiency with which we could detect microbial species in our samples using a widely used microbiome profiler, MetaPhlAn4 [46], relative to our RuMAG database. We were able to reliably detect more than two times as many species (2013 vs. 828 species) using our database relative to MetaPhlAn4. This both highlights the importance of comprehensive environment specific genome databases and allowed for significantly improved metagenomic resolution and analysis in this study.

### Microbial diversity and VFA biosynthetic potential of RuMAGs

Metagenomic read mapping against our cohort-specific genome database revealed a total of 2013 species-level RuMAGs across the two animals that were profiled, with 1857 and 1411 species detected in the control and treatment animal, respectively. Qualitatively, we observed that the 603 species of *Bacteroidota* detected were in relatively high abundance (each species accounting on average for 0.1 ± 0.3% of the observed relative abundance). Additionally, some selected species within the *Pseudomonadota*, *Verrucomicrobia*, and *Fibrobacteria* were also highly abundant (Fig. 1A). Alternatively, *Firmicutes* were represented by a large taxonomic diversity (1055 species) but each *Firmicute* species contributed considerably less relative abundance to communities on average (0.01 ± 0.05% average relative abundance per species).

To evaluate the potential of the bacterial rumen population for VFA production, we systematically profiled RuMAGs to look for genes involved in VFA biosynthesis (see “Materials and methods” section). The potential for acetate biosynthesis was widespread, with the phosphotransacetylase/acetate kinase (PTA/AK) pathway being the dominant route (Fig. 1A andFig. S2A). Alternative routes for acetate biosynthesis via the Acetyl-CoA (generating ADP) and Butyryl-CoA pathways were also detected specifically in *Bacteroidota*. Acetate biosynthesis through the Acetyl-CoA synthetase (ACS) route generating AMP was rare in RuMAGs, and the potential for propionate and butyrate biosynthesis was found to be clade-specific (Fig. 1A andFig. S2A). Propionate biosynthetic pathways were primarily found among the *Bacteroidota*, with the succinate-mediated pathway being the primary route. Alternative pathways for propionate biosynthesis were only detected in a limited number of RuMAGs and pathways for butyrate biosynthesis were primarily detected in subgroups of *Firmicutes* and *Bacteroidota*. Unlike RuMAGs encoding pathways for acetate and propionate production, we found that RuMAGs encoding butyrate production pathways frequently encoded multiple routes. Specifically, we noted the co-existence of lysine- and pyruvate-mediated butyrate pathways in *Bacteroidia* RuMAGs.

Taken together, the observed relative abundance of bacterial species and their VFA production potential suggest that *Bacteroidota* species likely play an important role in VFA production in these samples, particularly for butyrate biosynthesis.

### Metatranscriptomic profiling captures relevant bacterial and archaeal species

Transcriptome profiling of all 8 rumen fluid samples collected during this study identified transcripts from 1865 microbial RuMAGs across all samples, including 1784 and 1254 species in the control and treatment rumen microbiome respectively. However, most of the detected RuMAGs recruited only a small number of transcripts suggesting low transcriptional activity, with the majority of transcripts (73.2 ± 10.8%) mapping to a core set of 110 bacterial and 3 archaeal RuMAGs that were considered transcriptionally active (≥ 20% of their genes transcribed in at least one sample). These 113 species broadly encompass RuMAGs detected at high relative abundance in our metagenomic samples and spanned 7 bacterial and 1 archaeal phyla (Fig. 1 andFig. S2). In the control group, two RuMAGs belonging to the *Enterobacterales* and *Bacteroidales* were highly transcriptionally active, contributing 18.4 ± 16.5% and 11.1 ± 3.2% of all mapped metatranscriptome reads respectively. Alternatively in treatment samples, the 10 most abundant RuMAGs recruited on average a similar fraction (3.0 ± 1.1%) of metatranscriptome reads. Overall, a comparison between the rumen metatranscriptome profiles of treated and untreated animals found a statistically significant separation based on treatment (*p* value = 0.034; PERMANOVA) between these sample sets (Fig. S3).

### Asparagopsis armata treatment decreases archaeal abundance and transcription

Within the metagenomes, *Methanobrevibacter *sp. from the *Methanobacteriota* and *Methanomethylophilus *sp. from the *Thermoplasmatota* were the most abundant archaeal methanogens detected (Fig. 1B). Functional profiling of methanogenesis pathways across these two taxa confirmed that *Methanobrevibacter *sp. RuMAGs encoded the genes to perform hydrogenotrophic methanogenesis while *Methanomethylophilus *sp. RuMAGs encoded the genes necessary for methylotrophic methanogenesis, which involves methanol and methylated amine reduction (Fig. 1B). Our metagenomic data also showed that *Methanomethylophilus *sp. were the dominant members within the archaeal methanogen populations, accounting for 66.7% and 82.2% of the archaeal relative abundance in the control and treatment metagenomes respectively*.* Previous 16S rRNA amplicon based rumen surveys [47] suggested that hydrogenotrophic methanogens of *Methanobrevibacter *sp. dominate methanogenic rumen populations, accounting for ~ 74% of the 16S rRNA gene amplicons. This discrepancy in archaeal species abundance between studies may be due to the fact that *Methanobrevibacter *sp. possess 2–3 copies of the 16S rRNA gene, whereas *Methanomethylophilus *sp. possess 1 copy [48], leading to an overestimation of *Methanobrevibacter sp.* using 16S rRNA profiling methods*.* Alternatively, shotgun metagenomics is agnostic to uneven copy numbers across genomes when abundances are quantified using MAGs [49].

**Fig. 2:**
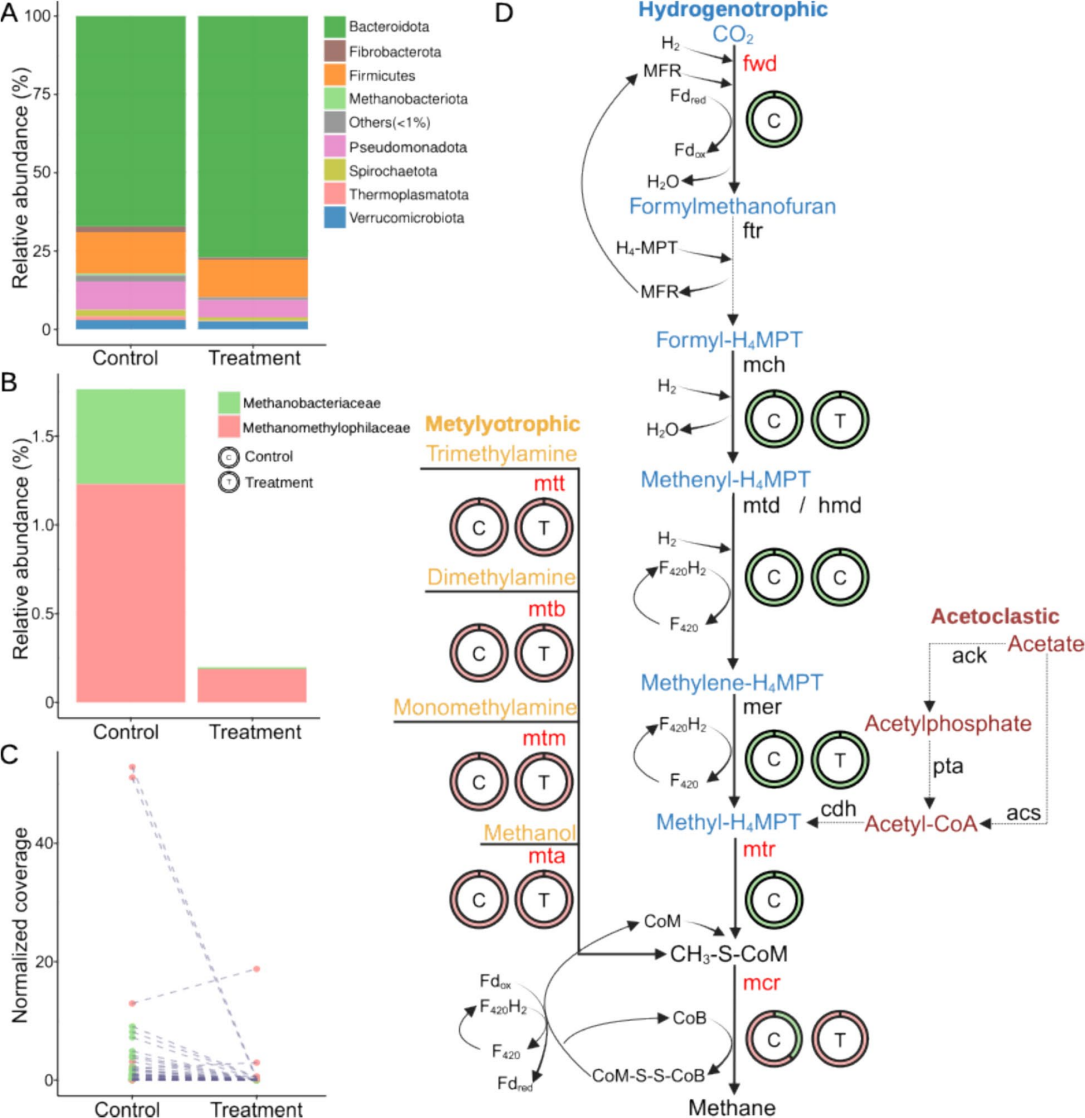
A. *armata* supplementation impacts on microbial abundance and methanogenic pathways. **A** Microbial relative abundance at the phylum level from metagenomic samples. Bacterial phyla whose relative abundance is < 1% are labeled as “Others.” **B** Archaeal relative abundance at the family level from metagenomic samples. **C** Coverage of individual archaeal RuMAGs between treated and control metagenomic samples. Lines connect the same genome detected in control and treatment samples. **D** Gene expression changes across 3 profiled methanogenic pathways, with the names of metabolic intermediates colored based on the type of methanogenic pathway (methylotrophic—yellow, hydrogenotrophic—blue, acetoclastic—red). Solid and dotted black lines indicate transcripts for an enzyme were or were not detected in samples, respectively. Enzyme names depicted in red indicate that at least one enzyme subunit was significantly downregulated (FDR ≤ 0.05) in treated samples (no subunit was upregulated). Enzyme names in black showed no statistically significant difference between samples. Circles show the relative contribution of archaeal families to the transcriptome reads of each enzyme in both control (C) and treated (T) samples

Metagenomic profiling revealed that treatment with *A*. *armata* resulted in a reorganization of the rumen microbiome including a qualitative increase of *Bacteroidota* and decrease of *Pseudomonadota* within the treated animal relative to the control animal (Fig. 2A). Moreover, there was a reorganization of the most abundant RuMAGs in the control and treatment rumen microbiomes (Fig. S4). There was a notable qualitative effect on archaeal populations, which decreased in abundance from 1.76% (control) to 0.02% (treatment) (Fig. 2B). In particular, *Methanobacteriaceae *sp. were relegated almost undetectable, and we observed that the relative abundance of almost all individually profiled archaeal genomes fell to near zero with exception of two *Methanomethylophilaceae *sp. genomes (Fig. 2C).

The qualitative reduction of archaeal populations, in samples treated with *A*. *armata*, paralleled a broad and significant decrease of transcripts associated with methanogenesis pathways (Fig. 2D). This included significant suppression of transcription of the formylmethanofuran dehydrogenase subunit A (fwdA; FDR = 0.046; log_2_FC = − 5.8; DESeq2) and the methyltransferase subunits A and H (mtrAH; FDR = 0.046/0.039; log_2_FC = − 5.3/− 5.6; DESeq2) and transcripts for the methyl coenzyme M reductase enzyme (mcrABG; FDR = 0.09/0.00001/0.039; log_2_FC = − 3.1/− 7.9/− 5.6; DESeq2). Additionally, while several genes involved in hydrogenotrophic methanogenesis were not detected as significantly downregulated, all exhibited decreases in transcript abundance in treated samples (Table S5). Consistent with our metagenomic analysis (Fig. 1B and Fig. S2B), transcripts for acetoclastic methanogenesis were not detected in our metatranscriptomic data (Fig. 2D).

### Hydrogenotrophic metabolism is activated in A. armata-treated animals

Roque et al. observed that the significant decrease in the production of CH_4_ per dry matter intake in animals that received *A*. *armata* was also coupled to a significant increase in H_2_ emissions [19] (Table S1). To evaluate how higher H_2_ partial pressures may impact rumen microbiome activity, we specifically assessed the expression of the catalytic subunit proteins for 38 known hydrogenase subgroups [50, 51] between treated and control samples.

**Fig. 3:**
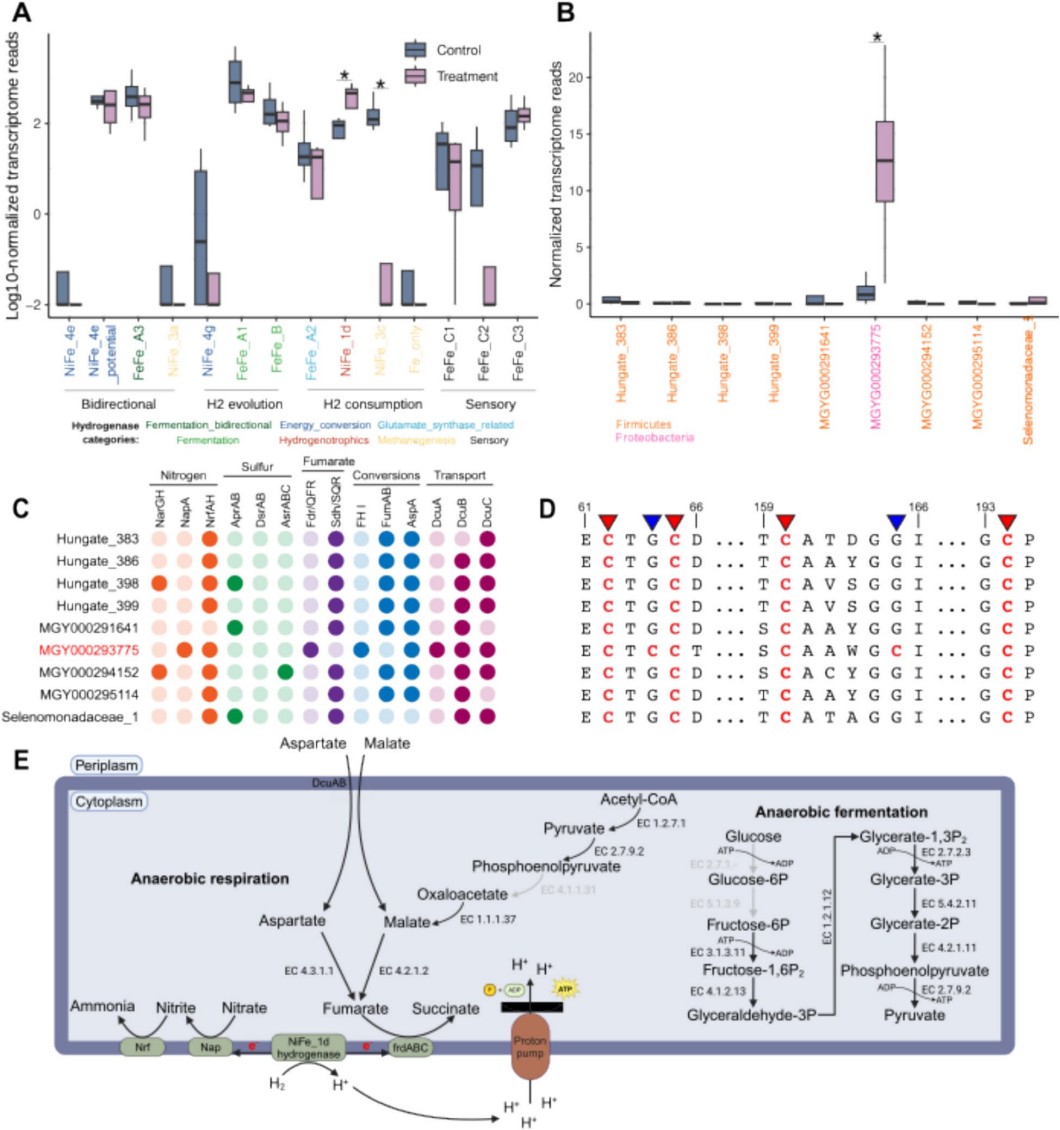
Effect of *Asparagopsis armata *supplementation on hydrogen metabolism and metabolic analysis of hydrogen utilizing RuMAGs. **A** Transcriptional changes of 14 hydrogenase families with detectable expression between control and treatment samples. The y-axis represents log_10_-normalized transcriptomic reads (per million reads) mapped to the catalytic subunit representatives of each hydrogenase subgroup detected in RuMAGs. Hydrogenase family names on the x-axis are colored and organized by the direction of hydrogen flow. Asterisks indicate significant differences in expression between treatment and control (FDR ≤ 0.05; DESeq2). **B** Normalized counts of transcriptome reads contributing to NiFe-1d expression in 9 species encoding and expressing this hydrogenase family. **C** Comparative genomic analysis of the 9 NiFe_1d encoding species focused on terminal electron accepting pathways. MGYG000293775 is colored red for easier readability. Potential pathways are organized by nitrogen metabolism (orange), sulfur metabolism (green), fumarate metabolism (purple), fumarate interconversions (blue), and metabolite transport (magenta). The presence of a function in a genome is indicated by a dark circle, while lack of function is indicated by a light circle. *NarGH* dissimilatory nitrate reductase, *NapA* periplasmic nitrate reductases, *NrfAH* ammonia-forming nitrite reductase, *AprA* adenylylsulfate reductase, *DsrAB* dissimilatory sulfite reductase, *AsrABC* anaerobic sulfite reductase, *Fdr*/*QFR* quinol:fumarate reductase, *Sdh*/*SQR* succinate dehydrogenase/succinate-ubiquinone oxioreductase, *FH I* fumarate hydratase class I, *FumAB* fumarate hydratase, *AspA* aspartate-ammonia lyase, *DcuABC* C4-dicarboxylate antiporters. **D** Portion of a multiple sequence alignment of NiFe_1d small subunit proteins from the 9 species in panel (**C**). Proteins are associated with species in the same order as shown in panel (**C**). This alignment depicts the proximal iron-sulfur cluster binding motif in the NiFe_1d small protein subunit. Metal binding cysteines are colored red in the alignment. The 4 cystines conserved across all proteins, which are typically involved in the formation of a 4Fe-4S cluster are highlighted by red triangles. The substitution of 2 cysteines with 2 glycines that differentiate MGYG000293775 from other species, enabling the formation of a 4Fe-3S cluster, are highlighted by blue arrows. Alignment positions are noted above the alignment. Also see Supplementary Datasets. **E** Metabolic reconstruction of potential energy generating pathways in MGYG000293775 focused on H_2_ coupled fumarate reduction. Black and gray arrows represent presence or absence of genes encoding reactions, respectively

Hydrogenase families associated with fermentative H_2_ evolution, specifically FeFe_A1 and FeFe_B, were actively and highly transcribed by rumen bacteria yet showed no significant differences in mean transcript levels between treated and untreated samples (Fig. 3A). We found that the majority of the FeFe_A1 family transcripts originated from only two RuMAGs, a *Succinivibrionaceae *sp. (MGYG000292509) and a *Ruminococcaceae *sp. (MGYG000291593) together contributing 78.3 ± 10.8% and 81.8 ± 2.2% of these transcripts on average in control and treatment samples, respectively (Fig. S5A). Similarly, FeFe_B family transcripts largely originated from a small number of RuMAGs including a *Treponematales *sp. (RuMAG_Treponema_1) contributing 46.3 ± 10.6% of these reads on average in control samples, and 4 *Bacteroidales* RuMAGs contributing 78.8 ± 25.3% of these reads on average in treated samples (Fig. S5B). Overall, these results suggest that transcription of H_2_ evolving hydrogenases is dominated by a small number of species in our samples and generally unaffected by treatment with *A*. *armata*.

We identified two hydrogenase families (i.e., NiFe_3c and NiFe_1d) associated with hydrogen consumption that significantly differed in overall expression between treatment and control samples. Furthermore, we observed that H_2_ consuming hydrogenases contributed a relatively larger fraction of transcripts to the total pool of hydrogenase transcripts in *A*.* armata* treated samples (control = 18.1 ± 7.3%; treatment = 36.8 ± 5.5%; Fig. S6A). The NiFe_3c hydrogenase family, which is involved in methanogenesis, was significantly downregulated (FDR = 0.004; log_2_FC = − 4.5; DESeq2) in treated samples (Fig. 3A). Alternatively, the NiFe_1d hydrogenase family, reported to be involved in hydrogenotrophic metabolism [51], was significantly upregulated (FDR = 0.04; log_2_FC = 2.1; DESeq2) in treated samples (Fig. 3A). Out of 9 genomes expressing NiFe_1d hydrogenases, we observed that transcripts were primarily derived from a single *Duodenibacillus *sp. RuMAG (MGYG000293775; control = 37.4 ± 39.8%; treatment = 95.7 ± 4.3%) within the *Pseudomonadota* (Fig. 3B). Our metagenomic analysis also showed that the relative abundance of MGYG000293775 increased by 58.3-fold between the single treated vs. control samples evaluated. Abundance and gene expression of MGYG000293775, a hitherto uncultured bacteria classified as a *Duodenibacillus *sp., prompted us to search for metabolic traits that may confer an advantage to this hydrogenotrophic microbe when competing for hydrogen as electron donor. We comparatively evaluated the potential of all 9 transcriptionally active, NiFe_1d encoding, hydrogenotrophic RuMAGs to couple hydrogen oxidation to several anaerobic terminal electron acceptors (Fig. 3C). Among these, 3 RuMAGs encoded genes for dissimilatory sulfur reduction, and 8 encoded genes for dissimilatory nitrogen reduction.

### Duodenibacillus sp. encodes advantageous energy generating pathways

All nine NiFe_1d encoding RuMAGs evaluated had at least some encoded capacity to couple hydrogen oxidation to terminal electron accepting processes (Fig. 3C). However, only 3 RuMAGs, including MGYG000293775, had the potential for complete reduction of nitrate to ammonia encoding both a nitrate reductase (NapA or NarG) and nitrite reductase (NrfA). Distinctively, MGYG000293775 was the only species encoding a periplasmic nitrate reductase (NapA), which is speculated to exhibit increased nitrate affinity [52], and a quinol:fumarate reductase (FdrA/QFR) capable of utilizing fumarate as a terminal electron acceptor and H_2_ as the corresponding electron donor [53, 54]. The succinate-ubiquinone oxidoreductase enzymes (SdhA/SQR) encoded in the other 8 RuMAGs preferentially oxidize succinate to fumarate, and do not typically enable the use of fumarate as a terminal electron acceptor [55–57]. Moreover, we observed that expression of all quinol:fumarate reductase subunits (FdrABC), nitrate reductase (NapA), and nitrite reductase (NrfA) in MGYG000293775 increased in treatment relative to control samples (Fig. S6B).

Given the important role of fumarate in propionate biosynthesis [58], free fumarate may be scarce in the rumen environment. A combination of fumarate and red seaweed supplementation might enable fumarate utilizing species to overcome this substrate limitation and redirect hydrogen toward propionate under methane-inhibiting conditions. In the human gut, where freely available fumarate is also limited, some species support H_2_/fumarate respiration through the import of C4-dicarboxylates (e.g., malate and aspartate) via C4-dicarboxylate membrane transporters and the subsequent conversion of the imported C4-dicarboxylates into fumarate, while simultaneously exporting succinate [59]. Accordingly, we evaluated if dcu genes encoding these C4-dicarboxylate/succinate antiporters were present in the 9 hydrogenotrophic RuMAGs evaluated. We found that MGYG000293775 encoded 8 C4-dicarboxylate antiporter (Dcu) genes, more than all other hydrogenotrophic genomes, including a DcuA variant that was absent from the other 8 RuMAGs (Fig. 3C). Moreover, we found that Dcu transporters in the MGYG000293775 genome were co-located with genes that may support H_2_/fumarate respiration by mediating the conversion of C4-dicarboxylates to fumarate (Table S6). This included the co-location of *dcu*A with an aspartate-ammonia lyase (*aspA*), the proximal co-location of *dcu*B (i.e., within 11 genes) from a fumarate hydratase (*FH I*), collectively enabling the conversion of both l-aspartate and l-malate into fumarate. While the other 8 hydrogenotrophic genomes evaluated encode aspA and fumA, these were not co-located with dcu transporters.

As sequence variation can impact the activity and oxygen tolerance of NiFe_1d hydrogenases [60, 61], we evaluated the sequence similarity of these hydrogenases across all 9 NiFe_1d encoding RuMAGs (Supplementary Datasets). We found that the essential L1 (xxRICGVCTxxH) and L2 (SFDPCLACxxH) motifs in the NiFe_1d large subunit were completely conserved across sequences from all 9 NiFe_1d encoding RuMAGs in our analysis. However, the NiFe_1d small subunit protein of MGYG000293775 was the only protein that contained 6 cysteines in the proximal iron-sulfur cluster motif (ECTxCC…xCAxxGC…GCP) compared to 4 cysteines (ECTxCx…xCAxxGx…GCP) observed in the other small subunit proteins (Fig. 3D and Supplementary Datasets). The presence of 6 cysteines enables the formation of an oxygen tolerant 4Fe-3S cluster, as opposed to the formation of a 4Fe-4S cluster when 4 cystines are present, and may have an impact on the enzymatic reaction rate of the NiFe_1d hydrogenase [50, 60]. Taken together, these findings show that MGYG000293775 possesses a multitude of metabolic traits (Fig. 3E) that may be potentially advantageous and provide a competitive edge relative to other hydrogenotrophic rumen microbes.

### A. armata treatment suppresses expression of carbon catabolic genes

**Fig. 4:**
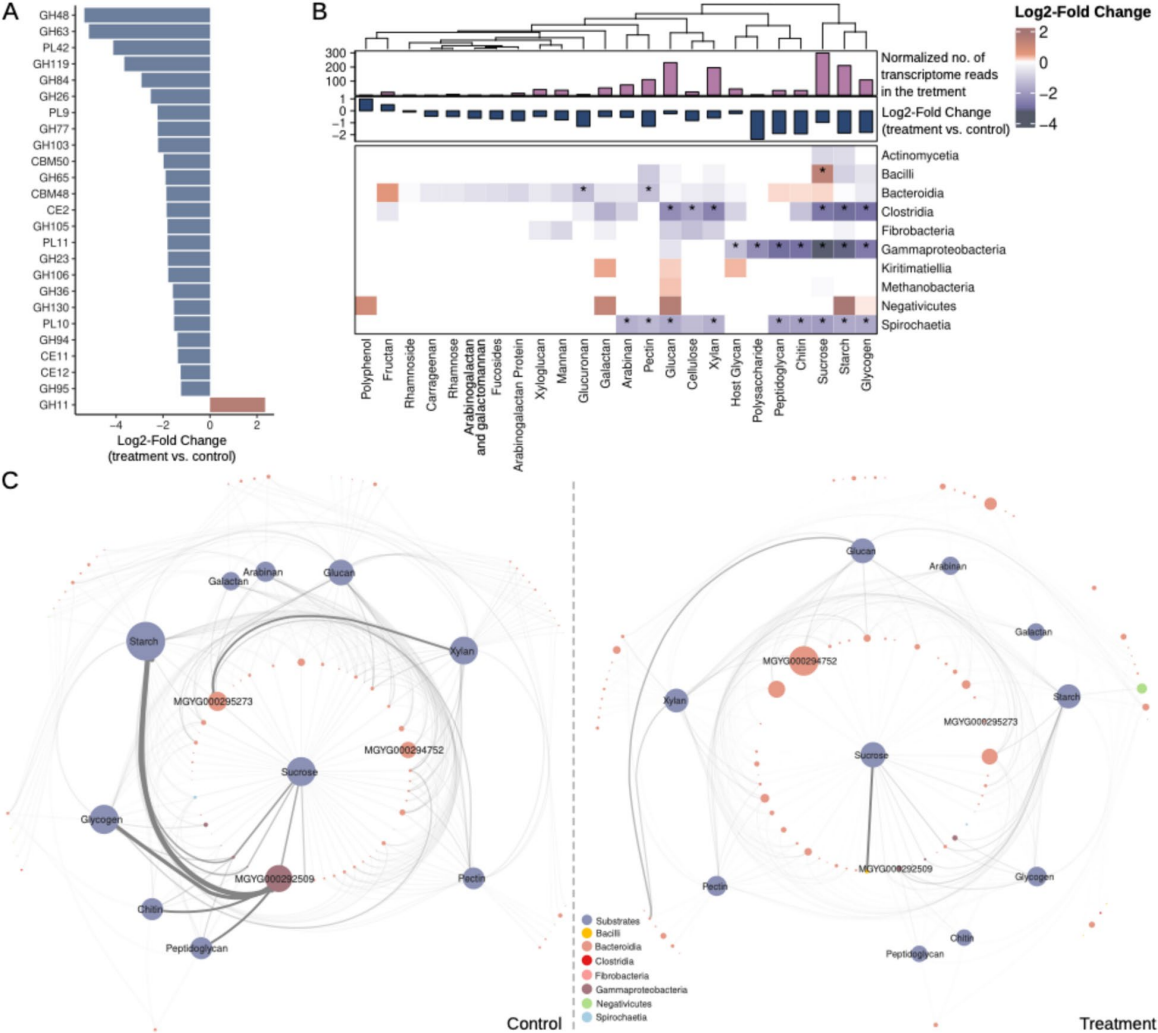
Effect of *A*. *armata *supplementation on carbohydrate metabolism.** A** Changes in expression of 25 CAZyme families with significant differential expression between control and treated samples. The x-axis shows log_2_-fold change from DESeq2 (FDR ≤ 0.05). The y-axis shows individual CAZyme families. Glycosyl transferases (GT) were omitted from the analysis. **B** Changes in expression of CAZYmes grouped by order-level microbial taxa and potential substrate. If a CAZyme family acted on more than one substrate, it was counted for both. The heatmap represents log_2_(treatment/control) change of normalized transcripts assigned to CAZymes degrading a specific substrate in each class. The bar plots above the heatmap show the total normalized number of transcripts (per million reads) assigned to CAZymes targeting each substrate (upper, purple), and log_2_(treatment/control) change of normalized transcripts assigned to specific substrates (lower, blue). **C** The top 250 RuMAGs and top 10 substrates, assessed by total CAZyme transcription in either control (left panel) or treatment (right panel) samples. Genomes (non-blue nodes) are connected to substrates (blue nodes) if they express CAZymes active on that substrate. The width of network edges denotes the expression level of CAZymes targeting a substrate. The size of the carbohydrate nodes represents the total normalized number of reads assigned to the CAZymes targeting the specific substrate. The size of the RuMAGs nodes represents the relative abundance in either the control or treatment sample measured by metagenomics

Syntrophic interactions between bacteria and methanogens have been reported [62], and in culture, H_2_ partial pressure has been shown to impact rumen bacterial cellulolytic activity [63]. Given the strong impact of *A*. *armata* treatment on the abundance and activity of rumen methanogens (Fig. 2B–D), we evaluated the expression of genes involved in the degradation of complex plant carbohydrates. Differential gene expression profiling revealed 25 carbohydrate active enzyme (CAZymes) families with significant expression differences between treated and untreated animals (Fig. 4A and Table S7). Of these 25 CAZyme families, all but one, CAZyme family GH11, were significantly downregulated in the microbiomes of ruminants that received *A*. *armata* (Fig. 4A). The CAZyme family most strongly downregulated was Glycoside Hydrolase Family 48 (FDR = 2.4e^−4^; Log_2_FC = − 5.35), which includes chitinase, cellobiohydrolase, and cellulase enzymes, known to significantly contribute to fiber degradation in the rumen [7, 64, 65]. Numerous other CAZyme families targeting plant polymers including hemicelluloses (GH26 and GH130) and pectins (PL9, PL11, and GH106) were also transcriptionally downregulated. Xylanases, belonging to CAZyme family GH11, were the sole GH family upregulated in the treatment group. GH11 transcripts were primarily derived from *Bacteroidota* and *Fibrobacterota* in control samples, and this shifted toward more dominant expression from *Bacteroidota* in treated samples (Fig. S7). Members of the GH11 family from *Fibrobacter succinogenes* were enriched in the metaproteome of the fiber adherent rumen microbiome [7] and expression levels of this family have been reported to be higher in feed efficient animals [66].

Next, we evaluated the relative contribution of the rumen microbiome, at class-level resolution, to the expression of CAZymes and their potential substrates (Fig. 4B). Transcripts from CAZymes with substrate specificity toward common plant biomass carbohydrates, including sucrose, starch, xylan, glucan, and pectin, were derived from microbes across numerous taxonomic classes, highlighting the overall importance of these CAZymes for rumen biomass degradation. However, the number of CAZyme transcripts declined across almost all substrate categories and taxonomic classes in the rumen microbiomes of animals fed an *A*. *armata* supplemented diet (Fig. 4B). Members of the *Bacteroidia*, *Clostridia*, *Gammaproteobacteria*, and *Spirochaetia* exhibited transcriptional activity across the largest set (≥ 7) of potential substrates. However, CAZyme transcripts primarily from *Clostridia*, *Gammaproteobacteria*, and *Spirochaetia* were significantly downregulated across most potential substrates in the rumen microbiomes subjected to *A*. *armata* (Fig. 4B). Despite the overall decrease in CAZyme expression across taxonomic groups in the presence of *A*. *armata*, the relative contribution of different taxonomic classes to substrate-specific CAZyme groups remained consistent (Fig. S8). Furthermore, CAZyme downregulation was minimal for *Bacteroidia* and not observed for *Fibrobacteria*, a taxonomic group that includes cellulolytic rumen bacteria such as *Fibrobacter succinogenes* S85 (*Fibrobacter succinogenes*).

The consistency of the relative contributions of class-level microbial lineages to CAZyme expression between treatment and control samples (Fig. S8) prompted us to investigate if there was a reorganization of CAZyme gene expression at the species level. In control samples, two RuMAGs from *Succinivibrionaceae* (MGYG000292509) and *Prevotella* (MGYG000295273) respectively displayed the highest CAZyme expression (Fig. 4C). These RuMAGs were also the two most abundant in the control sample we assessed with metagenomics. MGYG000292509 expressed CAZymes linked to starch, glycogen, peptidoglycan, and chitin degradation, while MGYG000295273 expressed CAZymes primarily linked to xylan degradation (Fig. 4C). In samples treated with *A*. *armata*, we observed significantly decreased CAZyme transcription from both RuMAGs. Moreover, a variety of other RuMAGs became the dominant contributors to CAZyme gene expression, and we observed a species level reorganization of CAZyme gene expression despite consistent class-level contributions (Fig. 4C and Fig. S8).

### The influence of A. armata on transcription of VFA biosynthesis genes

**Fig. 5:**
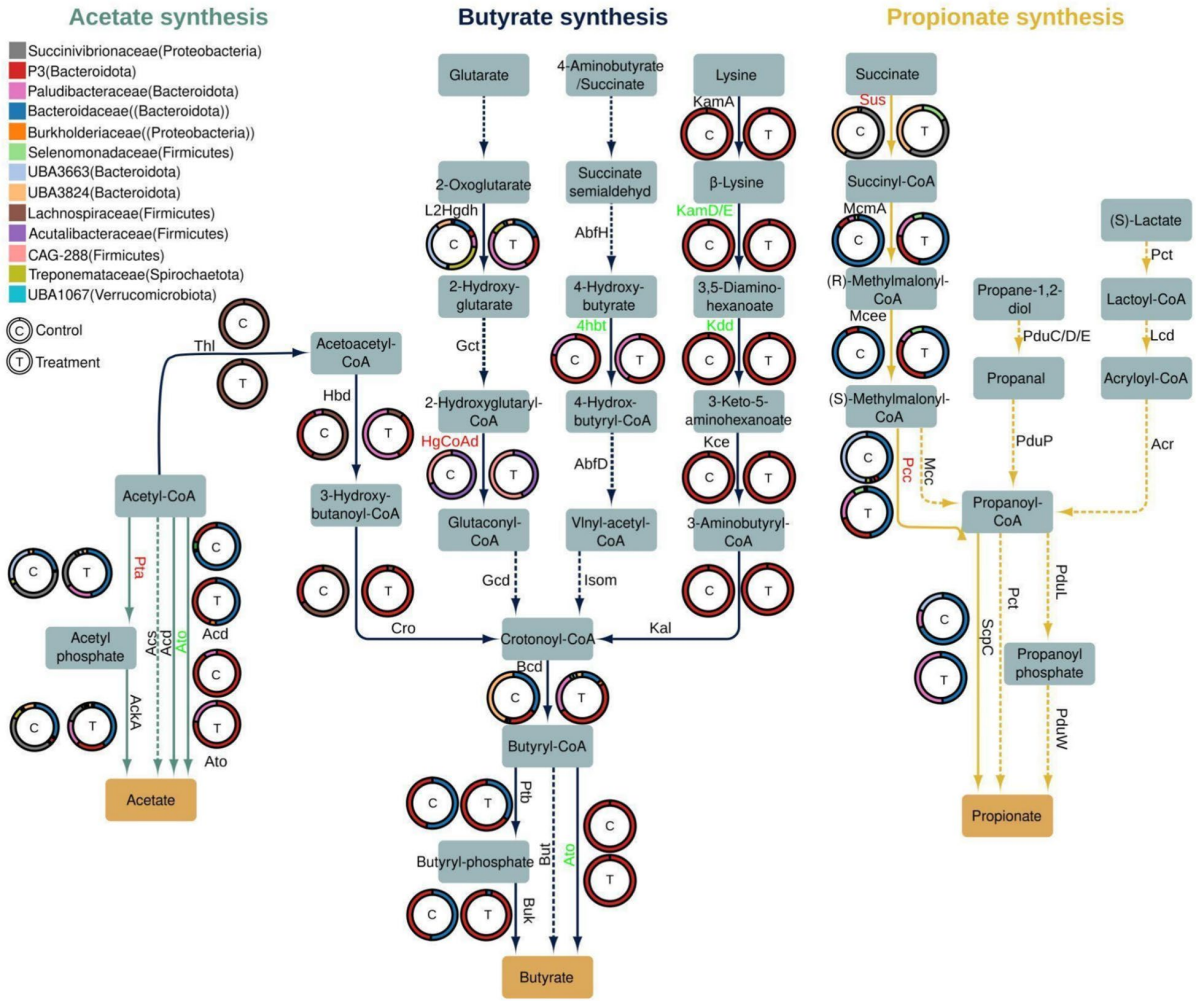
Effect of *A*. *armata* supplementation on transcription of volatile fatty acid biosynthesis pathways. Pathways and intermediates for acetate, butyrate, and propionate biosynthesis are shown. Arrows indicate individual reactions and gene names for protein products mediating each reaction are shown next to arrows. Solid and dashed arrows indicate that a gene product was or was not detected in our metatranscriptomic data, respectively. Arrows are colored based on the presence of reactions as part of acetate (teal), butyrate (blue), and propionate (yellow) biosynthesis. Gene names are depicted in green or red to indicate significant upregulation or downregulation (FDR ≤ 0.05; DESeq2) in the treatment condition, respectively. For reactions with multi-subunit enzymes to be considered up- or downregulated, at least one subunit had to be differentially expressed and no other subunit could have the opposite direction of expression. The rings present next to each gene product give the fraction of transcriptomic reads associated with genomes of different taxonomic families that are assigned to each enzyme in control (C) and treatment (T) samples. For taxonomic profiles the number of reads from all gene subunits were aggregated. Also see Table S8

Previous work has shown that supplementation with *Asparagopsis *sp. does not decrease VFA titers in vitro [67, 68] nor in vivo [37], and has no negative impact on animal feed conversion efficiency [19] (Table S1). To investigate the effect of *Asparagopsis *sp. supplementation on the transcription of prominent VFA biosynthetic pathways (e.g., acetate, butyrate, and propionate), we profiled the expression of the genes mediating the biosynthesis of these compounds (Fig. 5 and Table S8).

In the biosynthesis of acetate, we observed transcriptional changes in two biosynthetic routes, the *pta/ackA* (phosphate acetyl-transferase/acetate kinase) mediated route where acetyl phosphate is an intermediate, and the Acetyl-CoA to acetate route mediated by *ato* (butyryl-CoA:acetoacetate CoA transferase). The expression of *pta* was primarily associated with RuMAGs of the *Bacteroidaceae* and *Succinivibrionaceae* taxonomic families, and *pta* transcription was significantly downregulated (FDR = 2e^−6^; Log_2_FC = − 2.0; DESeq2) in the rumen microbiome subjected to *A*. *armata* (Fig. 5). Furthermore, we observed that in treated samples, *pta* transcripts from the *UBA3663* and *Paludibacteraceae* families contributed to a lower and higher fraction of overall pta transcripts, respectively. The expression of *ato* was primarily associated with RuMAGs of the *Paludibacteraceae* and *P3* taxonomic families, and *ato* transcription was significantly upregulated (FDR = 0.002; Log_2_FC = 2.5; DESeq2) in the rumen microbiome subjected to *A. armata* (Fig. 5). No major changes to the taxonomic distribution of *ato* transcripts was observed.

Transcripts for genes that mediate butyrate biosynthesis from 4 independent precursors, acetoacetyl-CoA, glutarate, 4-aminobutyrate/succinate, and lysine were detected in our samples (Fig. 5). In the lysine-derived butyrate pathway, we observed a significant increase in the transcription of genes for three steps, *kamDE* (β-lysine-5,6-aminomutase; FDR = 0.0036; log_2_FC = 1.7, DESeq1), *kdd* (3,5-diaminohexanoate dehydrogenase; FDR = 0.0036; log_2_FC = 1.99, DESeq2), and *ato* (acetyl-CoA acyltransferase; FDR = 0.0003; log_2_FC = 2.4; DESeq2) in treated samples (Fig. 5). Due to *ato* functioning in both acetate biosynthesis and the lysine-dependent butyrate biosynthesis pathways, respectively [69, 70], we evaluated *ato* transcripts associated with the lysine-dependent butyrate pathway by only including those from organisms that expressed full sets of genes for this pathway (i.e., *kamA*, *kamDE*, *kdd*, *kce*, *kal*, *bcd*, and *ato*).

Expression of genes associated with propionate biosynthesis was exclusively detected for the succinate-mediated route (Fig. 5). The expression of both *sus* (Succinyl-CoA synthetase; FDR = 0.001; log_2_FC = − 2.6; DESeq2) and *pcc* (propionyl-CoA carboxylase; FDR = 0.039; log_2_FC = − 0.9; DESeq2) were significantly decreased in microbiomes of the treatment group. Taken together, we found that genes mediating VFA biosynthesis were primarily expressed from RuMAGs in the phylum *Bacteroidota*, and that while most detectable genes for VFA biosynthesis were not differentially expressed, a limited set of potentially important transcripts were differentially expressed under methanogenesis suppression.

## Discussion

### Overview

Despite the effort toward developing advanced strategies for curbing the emissions of methane (CH_4_) from cattle [8, 71], these interventions often only lead to a partial reduction of CH_4_ emissions [8, 72] and fail to reroute methanogenic substrates toward alternative metabolites [32], which could enhance the productivity of the ruminant animal [9]. Developing approaches for targeted and lasting modulation of the rumen microbiome, in a manner that both diminishes CH_4_ output and bolsters animal production efficiency, necessitates a detailed microbiome understanding of this phylogenetically complex [47] and metabolically interconnected environment [73, 74]. This includes insights into the complex interactions among its diverse metabolic guilds and their collective response to the suppression or loss of methanogenic activity [75]. To further these goals, we performed a species-level metagenomic and metatranscriptomic evaluation of the effect of *A*. *armata*-supplemented feeding on the rumen microbiome. Our research revealed a pronounced reduction in both the abundance of methanogens and their transcriptional activity, widespread decrease in the transcription of CAZymes and the initiation of hydrogenotrophic pathways alongside the identification of species-specific metabolic characteristics that may offer a competitive edge to specific hydrogenotrophic species within the rumen ecosystem.

### Enduring deficiencies in rumen metagenome assembled genomes

Interrogation of the rumen microbiome at species-level resolution has been bolstered using genomes from cultured rumen microorganisms [43] and rumen metagenome assembled genomes (RUMAGs) [43, 44, 65, 76]. Despite the existence of several rumen-specific genome databases [43–45], we observed that microbial diversity in our samples remained underrepresented, with read mapping rates ranging from 1 to 18% using these existing databases (Table S4). This suggests that rumen microbiomes, even from cattle housed in industrialized countries and subjected to more standardized farming practices, still contain a large and unexplored genomic diversity, with bacterial groups in particular Likely remaining under-sampled. Despite the Limited metagenomic sampling of this work, our newly recovered genomes contributed 32 novel species to an integrated database of 3119 bacterial and 61 archaeal species. Importantly, we observed that the integration of multiple databases increased our ability to map metagenomic reads (Table S4) and enabled the interrogation of VFA biosynthetic potential across currently available rumen bacterial species (Fig. 1A and S2A), which to our knowledge has not previously been done at this scale. An important insight from this analysis is that rumen bacteria utilize numerous pathways for VFA biosynthesis, with a taxonomically diverse set of species across the phylum *Bacteriodota* encoding most of these pathways (Fig. 1A and S2A). Furthermore, metatranscriptomic analysis indicated that *Bacteriodota* were also the most active propionate, butyrate, and acetate producers in the rumen samples we evaluated (Fig. 5). Nonetheless, expanding databases of rumen-associated genomes will be essential in future research, as current collections are likely still incomplete.

### Effects on methanogenic archaea

In our study, *Methanomethylophilus* sp. and *Methanobrevibacter *sp. (Fig. 1B) were identified as the dominant methanogens, which aligns with previous work [47]. While *Methanobrevibacter *sp. have been traditionally reported as the more prevalent methanogen, constituting up to 74% of rumen archaeal populations [47], our analysis revealed a predominance of *Methanomethylophilus *sp. While this deviation may be an artifact of our small metagenomic sample size (*n* = 2), it may also be attributed to a difference in 16S rRNA gene copy number in their genomes, as *Methanobrevibacter *sp. can harbor up to three 16S rRNA copies, versus a single copy in *Methanomethylophilus *sp. [48]. These differences can only be detected and taken into consideration when determining abundances using whole genomes, which underscores the importance of using genome-resolved methods. Whereas traditional 16S rRNA amplicon sequencing remains a valuable tool to assay large numbers of samples and provide an overview of the makeup and changes to microbiome compositions, it is more limited in its ability to quantify methanogenic archaea, given the latter’s susceptibility to gene copy number variations [77]. *Methanomethylophilus *sp. represent a relatively novel and less understood group of methanogens [78] and their level of abundance in this study suggests that these, and other less studied microbes, could play a significant role in rumen methane emissions in some cattle populations. Our findings, based on metagenomic and metatranscriptomic data (Fig. 2) that are capable of detecting these less studied methanogens, indicate that *A*. *armata* supplementation can effectively suppress both hydrogenotrophic and methylotrophic methanogenesis, concurrently reducing the populations of both *Methanomethylophilus *sp. and *Methanobrevibacter *sp. (Fig. 2B, C). The decrease in methanogen abundance and activity (Fig. 2), paired with a rise in hydrogen production (Table S1), aligns with the established theory that methanogens are the most important hydrogen sink in the rumen [10]. The broader responses of the rumen microbiome to the loss of this hydrogen sink, on the other hand, remain poorly characterized.

### Effects on hydrogen metabolism and microbial competition

Here, we found that suppression of the methanogenic hydrogen sink led to targeted shifts in hydrogen metabolism. While the expression of fermentative H₂-evolving and bi-directional hydrogenases remained largely unchanged, there was a notable increase in the community-level expression of the H₂-consuming NiFe_1d hydrogenase (Fig. 3A). Among nine transcriptionally active species expressing NiFe_1d hydrogenases, MGYG000293775, classified as a yet unnamed species of *Duodenibacillus*, dominated in both activity and abundance when methanogenesis was suppressed (Fig. 3B). Addressing enteric methane emissions by diverting hydrogen from methanogenesis toward alternative pathways necessitates understanding the competitive interactions among hydrogenotrophic organisms [79] and MGYG000293775 has metabolic capacity that may provide advantages over other hydrogenotrophs. Notably, its flexible capacity for energy generation via anaerobic respiration, coupling hydrogen oxidation to the complete reduction of nitrate to ammonia or reduction of fumarate to succinate (Fig. 3C andE), might allow MGYG000293775 to outcompete other rumen hydrogenotrophs under methane suppression. Although other hydrogenotrophic rumen metagenome-assembled genomes (RuMAGs) encode anaerobic respiratory capabilities, the quinol:fumarate reductase (fdrA/FQR) was unique to MGYG000293775 (Fig. 3C). Moreover, while nitrate and sulfate have higher free energy potential as terminal electron acceptors [80, 81], their concentrations are typically low in the ruminant diet [21, 82], and likely do not play a major role under the conditions evaluated. Furthermore, the potential for MGYG000293775 to obtain fumarate via DcuABC mediated l-aspartate/l-malate import and succinate export [59] enables MGYG000293775 to utilize additional rumen metabolites for H_2_/fumarate respiration. These mechanisms, known to confer a growth advantage in other anaerobic systems [59, 83], may provide MGYG000293775 with a competitive advantage over other hydrogenotrophic rumen microbes, and could be potentially exploited in novel approaches for rumen microbiome modulation.

### Effects on the carbohydrate-active enzyme profiles

While it is well established that excess hydrogen inhibits some of the major cellulolytic rumen bacteria, and therefore could impact the overall degradation of complex carbohydrates in the rumen ecosystem [84], very little is known about the cellulolytic bacteria that are less sensitive to increased hydrogen and the repertoire of carbohydrate active enzyme (CAZymes) they employ under these conditions. Investigating rumen CAZyme expression profiles in our analysis revealed a widespread down-regulation of CAZyme families in *A*. *armata* treated animals. This downregulation was observed at the CAZyme family level (Fig. 4A), across most potential CAZyme substrates, and broadly across microbial taxonomic groups expressing these enzymes (Fig. 4B). Downregulation of CAZyme expression was especially prominent across genomes of the *Clostridia*, whereas significant downregulation of CAZymes in the *Bacteroidia* was limited to those involved in glucuronan and pectin metabolism (Figs. 4B and S8). The observed downregulation of microbial CAZymes did not negatively impact animal feed efficiency (Table S1), and notably the single CAZyme family with a significant increase in expression (GH11) has been reported as more highly expressed in feed-efficient animals [66]. However, due to the use of a standardized feed across all animals, we could not evaluate how changes in CAZyme expression may impact the adaptability to different feed types. Nonetheless, these findings may indicate that specific and important fibrolytic groups, such as the *Bacteroidia* and *Fibrobacteria*, may be generally more resistant to increased concentrations of hydrogen in the rumen, and that those robust bacteria play a more prominent role in biomass degradation under seaweed-induced inhibition of methanogenesis.

### Unresolved issues and future opportunities

Although VFAs were not directly quantified in this study, we observed a reduction in the expression of succinyl-CoA synthetase (Sus) and propionyl-CoA carboxylase (Pcc) in samples treated with *A*. *armata* (Fig. 5). This downregulation of propionate biosynthetic genes may reflect feedback inhibition, consistent with a scenario in which elevated propionate levels suppress the enzymes involved in its own synthesis [84]. Alternatively, a corresponding decrease in acetate availability would align with the observed upregulation of acetate biosynthetic enzymes (Fig. 5). Thus, while promising mechanisms for redirection and competition for hydrogen have been identified in MGYG000293775, the *A*. *armata* induced changes to VFA and CAZyme gene expression on rumen metabolite pools, and their impact on animal efficiency, requires further investigation.

A key question in developing effective methane mitigation strategies will be how to leverage microbiome adaptations enabling competitive hydrogen consumption, while maintaining comprehensive and efficient utilization of feed carbohydrates by the host animal [86]. This must also be done with consideration for the functional redundancies within the diverse rumen microbiome and its genomic landscape, such that rumen microbiome alterations can remain stable, competitive, and effective. Answers to outstanding questions may be found through rumen microbiome studies that employ in vitro rumen systems, such as ANKOM units or the RUSITEC [85] and through extended longitudinal animal trials during which significantly larger sample sets are collected and subjected to genomic, transcriptomic, and metabolomic approaches.

## Conclusions

We utilized a combination of metagenomics and metatranscriptomics to identify MGYG000293775, an uncultured rumen bacterium classified as a member of the genus *Duodenibacillus*, as a potential key driver in redirecting excess rumen H_2_ toward the conversion of fumarate to succinate, a precursor for propionate synthesis. This finding renders this *Duodenibacillus *sp. MGYG000293775 as a potential target for genome engineering toward advanced methane mitigation strategies. Isolating and cultivating the *Duodenibacillus *sp. identified in this work, and rumen hydrogenotrophic species more broadly, will be an important step toward our ability to mechanistically characterize, genetically modify, and potentially engineer these species to compete with hydrogenotrophic methanogens.

While providing first insights into the molecular response of the rumen microbiome and potential alternative pathways through which H_2_ can be redirected away from CH_4_, our work presented here has some limitations that should be addressed by conducting additional animal experiments in the future. Specifically, an increased number of animals and comparative analysis at multiple time points to capture the impacts of methanogenesis suppression, such as significantly increased hydrogen levels in the rumen, over extended time should be considered. Further work should also take into consideration the possibility of changes in the microbial response depending on the lactation state, age, and parities of the animals that receive *A*. *armata* supplemented feed. Despite the limitations of the work presented here, it still provides a valuable first look at the response of the rumen microbiome to red seaweed at gene expression resolution. This highlights the value and importance of expanding rumen microbiome studies that employ methanogenesis inhibitors, across time, locations, and breed, as well as utilizing the latest omics techniques to obtain a more detailed and finely nuanced understanding of the rumen microbiome.

## Materials and methods

### Experimental design and setup

Details of the animal trial, including details on how methane production was quantified, have been described previously by Roque and colleagues [19]. In brief, three sets of four Holstein cows (total *n* = 12) were randomly assigned to one of three treatment groups (control group: basal diet; low dose group: basal diet + 0.5% OM *Asparagopsis armata*; high dose group: basal diet + 1% OM *A*. *armata*), then fed the allocated diet for 14 days during which milk production and components, dry matter intake (DMI), body weight (BW), feed conversion efficiency (FCE), CH_4_, carbon dioxide (CO_2_), and hydrogen (H_2_) production were recorded. After the 2-week feeding period ended, cows were fed the basal diet for 7 days (washout period), before treatments were randomly reassigned to a different set of cows [86]. This ensured to expose all 12 cows to each of the three treatment groups for a 14-day period. For the work described here, rumen fluid was collected during the last day of the last period of experiment from four animals in the control group (fed basal diet) and from four animals in the high dose (fed basal diet with 1% OM *A*. *armata*) group. An overview of the experimental design is outlined in Fig. S1. The chemical composition of the basal diet and *Asparagopsis armata* is provided in Supplementary Table S11. Additional information on the animal trial can be found in Roque et al. [19].

### Sample collection and preparation

Two hours after feeding, when methane production as well as digestion of feed and feed supplements have been suggested to peak [87–89], cows were moved to a head gate where rumen fluid was collected from each cow in the control and high-dose group, resulting in eight fluid samples in total, using an oral stomach tube technique [90]. An oral-ruminal stomach tube fitted with a perforated brass probe head (Anscitech Co Ltd. Wuhan, China) was inserted through the mouth and into the rumen at an insertion depth of 200 cm to ensure that samples were collected from the central portion of the rumen, providing for less variability between samples [91]. During collection, the first 500 mL of sample was discarded to limit saliva contamination [92]. Approximately 200 mL of rumen fluid was captured in a 1 gallon prewarmed, insulated canister (Thermos, Schaumburg, IL) and transported immediately to the laboratory. Rumen fluid was flash frozen in liquid nitrogen and then stored at − 80 °C in 100 mL sterile centrifuge tubes to prevent sample degradation and to avoid changes in DNA and RNA profiles, after the rumen fluid was strained through four layers of cheesecloth. Rumen samples were stored at − 80 °C for no longer than 30 days prior to DNA and RNA extraction.

### DNA/RNA extraction and sequencing

One rumen fluid sample was randomly picked from each of the control and high-dose groups for metagenomic sequencing. DNA extraction was performed using the FastDNA SPIN Kit for Soil (MP Biomedicals, Solon, OH) with ~ 500 mg of sample according to the manufacturer’s protocol. DNA was subsequently purified with a Monarch PCR & DNA Cleanup Kit (New England Biolabs, Ipswich, MA) following the manufacturer’s instructions. Extracted DNA from each sample was diluted to a standard concentration of 20 ng/µL in a volume of 20ul giving a final DNA mass of 400 ng per sample. DNA was then stored at − 20 °C until shipped and was further processed for DNA shotgun sequencing at the DOE Joint Genome Institute (Berkeley, CA). Metagenomic sequencing was performed on an Illumina NovaSeq sequencer using NovaSeq XP V1 reagent kits, S4 flowcell, following a 2 × 151 indexed run recipe.

To obtain RNA, approximately 1 mL of eight frozen rumen fluid samples (*n* = 4 independent samples from control group and *n* = 4 independent samples from high dose group) were thawed and homogenized using an 18-gauge needle and syringe. RNA was then extracted and purified using the PureLink RNA Mini Kit (Invitrogen, San Diego, CA). After purification, the samples were then treated with DNA-Free Kit DNase treatment and removal (Invitrogen, Waltham, Massachusetts, USA) at room temperature for 15 min. RNA was quantified using a Bioanalyzer (Agilent, Santa Clara, CA) and RNA quality was evaluated using a sodium hypochlorite (6%) agarose gel as described previously [93]. All RNA samples were equally split in half then subjected to two different ribosomal RNA removal techniques [Ribo-Zero(TM) rRNA Removal Kit (Illumina, San Diego, CA, USA) and QIAseq FastSelect rRNA Removal Kit (QIAGEN, Germantown, MD, USA)]. Both RNA sample quantities were determined using Qubit 3.0 Fluorometer (Invitrogen, Waltham, Massachusetts, USA). Stranded cDNA libraries were generated using the Illumina Truseq Stranded mRNA Library Prep kit. The rRNA depleted RNA was fragmented and reversed transcribed using random hexamers and SSII (Invitrogen, Waltham, MA, USA) followed by second strand synthesis. The fragmented cDNA was treated with end-pair, A-tailing, adapter Ligation, and 8 cycles of PCR. The prepared libraries were quantified using KAPA Biosystems’ next-generation sequencing Library qPCR kit and run on a Roche LightCycler 480 real-time PCR instrument. Sequencing of the flowcell was performed on a Illumina NovaSeq sequencer using NovaSeq XP V1 reagent kits, S4 flowcell, following a 2 × 151 indexed run recipe.

### Recovery of rumen metagenome-assembled genomes

The two metagenomes were subject to quality control with FastQC (https://github.com/s-andrews/FastQC). The adaptors and PhiX contaminants were detected and trimmed by bbMap (https://sourceforge.net/projects/bbmap/) followed by quality trimming with sickle [94] with default parameters. The high-quality reads were assembled with IDBA (–mink 30 –maxk 150 –step 10) [95]. IDBA-UD mink, maxk, and step parameters were modified based on the approach of Diamond et al. [96]. The assembled contigs longer than 5000 bp were extended with COBRA [97] by the identification of overlapped kmers at both 5′ and 3′ terminus among all the contigs and consensus coverages across the joint contigs which was estimated by mapping the reads against the contigs with bbMap. The contig coverage was calculated using the jgi_summarize_bam_contig_depths script from the MetaBAT2 package [98].

In order to recover the MAGs from the contigs, cross-mapping of the two sets of metagenomic reads against the two assemblies (contigs ≥ 2.5 kb) was performed with SNAP [99] and the contig coverage was calculated by jgi_summarize_bam_contig_depths. The coverage file and assemblies were used to bin assembled contigs into genome bins (MAGs) using concoct [100], maxbin2 [101], metabinner [102], metabat2 [98], and vamb [103]. To remove the redundancy of MAGs and pick up the best-quality MAGs among different binners, DASTool [104] was deployed (–score_threshold 0.3), which resulted in 226 non-dereplicated MAGs across the two samples analyzed using metagenomics.

### Construction of non-redundant rumen prokaryotic RuMAG database

We identified additional microbial genomes from rumen samples from publicly available databases and integrated them with the MAGs recovered in this study. This included 457 isolate genomes from the Huntage1000 collection [43], 4941 MAGs from [44], and 5578 genomes from the Mgnify database [45]. Genomes from each set were individually de-replicated at the species level with dRep [105] (–S_algorithm ANImf -sa 0.95 -nc 0.10 -comp 60 -con 10). This step resulted in 345, 2177, 2729, and 72 non-redundant species-level genome representatives (ANI ≥ 95%) from the Hungate1000, RUGs, Mgnify, and MAGs from this study, respectively. Subsequently, the species-level representative genomes from each database were integrated and de-replicated at the species-level (ANI ≥ 95%) with dRep a second time to generate a final comprehensive database of 3180 non-redundant species-level representative genomes. We chose to evaluate taxonomy at the level of species (ANI ≥ 95%), as a precise ANI cutoff for strain level definition is not widely agreed upon. The taxonomic classification of the genomes was conducted with GTDB-TK [106]. To visualize the phylogenetic relationships among the genomes, the bacterial and archaeal trees were constructed with GToTree [107] by designating the bacterial and archaeal single-copy marker gene sets separately and trees were visualized with iTol [108].

### Functional annotations of RuMAGs

We employed several different approaches for genome functional annotation. KofamScan [109] (KofamKOALA; -T 0.75; Accessed August 2023) and eggNOG-mapper v2 (Accessed November 2023) [110] were used for general functional annotation of genomes. If multiple HMM profiles from KofamScan matched a gene, only the highest scoring hit was retained. Carbohydrate-active enzymes were predicted by using dbCAN3 [111]. Custom hidden Markov models (HMMs) were generated for hydrogenase classification. Briefly, the reference sequences from each group of hydrogenase within HydDB [51] were retrieved and multiple alignment was performed separately for each group of protein sequences using ClustalO [112]. This included major hydrogenase groups FeFe-1, FeFe-2, FeFe-3, FeFe-4, NiFe-A, NiFe-B, and NiFe-C. HMM models were constructed from each protein sequence alignment using the hmmbuild function of the HMMER3 package [113]. Reference sequences from all groups were scored against the resulting HMM models with hmmsearch, and HMM score thresholds for identifying group membership were set as 75% of the score from the lowest scoring reference sequence known to belong to the respective group, as described in Vital et al. [70]. Both an *E* value < 1e^−15^ and score threshold were used to filter the results when searching genomes against the hydrogenase HMM models with hmmsearch. To better annotate the predicted hydrogenases into well-defined subgroups (e.g., NiFe_1a, NiFe_1b, NiFe_1c), manual phylogenomic annotation was deployed. The predicted hydrogenases and reference hydrogenases from the same group were integrated and aligned with ClustalO. Phylogenetic trees were constructed using Fasttree [114]. The node of the last common ancestor for each hydrogenase subgroup was identified if the descendent nodes of the common ancestor harboring all the reference hydrogenases from that subgroup and all the descendent nodes apart from the reference hydrogenase nodes within that clade were assigned to the same hydrogenase subgroup as the reference hydrogenases. This well classified the subgroups for NiFe-1, NiFe-2, NiFe-3, NiFe-4, and FeFe-C. A set of NiFe-4 hydrogenase closest to NiFe-4e were found that did not form a clade with known references, and such were labeled as “NiFe-4e-potential” (Fig. S9). For FeFe-A hydrogenases, FeFe-A2 and FeFe-A4 could be classified on the tree but FeFe-A1 and A3 could not be well separated. FeFe-A3 hydrogenases have been reported to be coupled with NADH-dependent oxidoreductases [50], so the flanking genes of those unclassified FeFe-hydrogenases were extracted and the hydrogenases accompanied by NADH-dependent dehydrogenases or reductases were assigned to FeFe-A3 with the remainder assigned to FeFe-A1. For Fe-only hydrogenase, there are only 25 reference sequences in HyDB and therefore we did not construct HMM models for this class. Rather, we used KO annotation to identify Fe-only hydrogenases in our genomes since the 25 reference sequences all belong to KO entry K13942 in the KEGG database. The list of potential reductases receiving the electrons from hydrogen can be found in Table S9.

### Identification of methanogenesis and VFA synthesis pathways

The enzymes involved in methanogenesis pathways from methanol, acetate, methylamine and CO_2_, acetate, and propionate biosynthetic pathways were identified based on literature search and the corresponding KOs for these reactions were retrieved from the KEGG database [115]. For acetate and propionate biosynthesis pathways, we retrieved the corresponding KOs from the literature [116–119]. For butyrate biosynthesis, we leveraged the hmm model introduced here [70] to identify the corresponding genes. For each reaction, if one of the subunits is present in the genome, we assume this genome is capable of catalyzing this reaction. For the presence of a complete biosynthetic pathway, we only allow 2 missing reactions maximum. The rule of co-localization of butyrate biosynthesis-related genes was not applied here because of the fragmented scaffolds in MAGs. For a detailed description of the KO list involved in methanogenesis and VFA synthesis and criteria used to define the presence of a specific pathway, please refer to Table S10.

### Calculation of metagenomic RuMAG coverage

To calculate the genome coverage, we mapped the reads from metagenomic samples against our comprehensive rumen metagenome-assembled genomes (RuMAGs) database using bowtie2 [120]. Subsequently, the sample-specific coverage of individual RuMAGs was calculated using CoverM (–min-read-percent-identity 0.95 –min-read-aligned-length 50). To filter out the spuriously detected RuMAGs due to ambiguous alignment against regions conserved among different RuMAGs, we calculated the expected breadth of a genome as a function of its coverage with the following formula [121]:

Equation 1:

1 $$\mathrm{Expected}\;\mathrm{Breadth}\;=\;1\;-\;\exp\left(-0.883\;\ast\;\mathrm{Genome}\;\mathrm{Coverage}\right)$$

Equation 2:

2 $$\mathrm{Breadth}\;\mathrm{Deviation}\;=\;{\mathrm{Log}}_2\left(\mathrm{Observed}\;\mathrm{Breadth}/\mathrm{Expected}\;\mathrm{Breadth}\right)$$

The coverage of any genome from a single sample where < 100 reads were mapped to the genome or where the value Breadth Deviation < − 1 was changed to zero in our analysis and was considered not conclusively identified as present in the sample.

### Metatranscriptomic data analysis

The metatranscriptomic reads were subject to quality trimming in the same way as metagenomic reads as described above. rRNA reads were detected and depleted with sortmerna [122]. The reads generated from libraries prepared with different rRNA depletion methods were combined, given the fact that they should capture highly consistent transcripts (Fig. S10). To profile the transcript expression level, reads were mapped against the predicted ORFs in each genome with bowtie2 [120]. Only mapped reads with a MAPQ score ≥ 35 were retained. Expression levels were calculated by CoverM (–min-read-percent-identity 0.97 –min-covered-fraction 0.95, https://github.com/wwood/CoverM) and only genes covered by at least 5 reads were retained. To Limit evaluating large numbers of RuMAGs that had very low levels of transcriptional activity, RuMAGs were only retained for downstream transcriptional analysis if they were found to be transcriptionally active in at least 1 of 8 transcriptome samples. We operationally defined transcriptionally active as having ≥ 20% of genes within a RuMAG covered by transcripts in at least one sample. Following this filtering, the majority of transcripts (73.2 ± 10.8% per sample) mapped to a core set of 113 RuMAGs. Differential expression analysis was conducted with the DESeq2 package in R [123]. All genes from RuMAGs that were considered transcriptionally active were aggregated into a single table and mapped read counts were compared between control (*n* = 4 samples) and *A*. *armata* treated (*n* = 4 samples) animals. The *p* values for each gene or group of genes evaluated using DESeq2 were corrected using false discovery rate (FDR) with an FDR ≤ 0.05 considered significant. The variance stabilizing transformed expression profile produced by DESeq2 was used to conduct principal component analysis using the prcomp function in R. To assess the differences in overall transcriptome profiles between control and treatment samples, PERMANOVA was conducted based on *Bray Curtis* dissimilarity of the transformed expression profile with VEGAN package [124]. PERMANOVA was run using 9999 permutations and *p* values ≤ 0.05 were considered significant

## Supplementary Information

Supplementary Material 1.

Supplementary Material 2.

## Data Availability

Raw metagenomic and transcriptomic data used in this study are available from the JGI Genome Portal (https://genome.jgi.doe.gov/portal/). JGI Project ID numbers for each sample can be found in Table S2. De-replicated genome bins are available at https://zenodo.org/records/13786122.
